# Identification of metabolic reprogramming-associated biomarkers in endometriosis through integrated bioinformatics analysis

**DOI:** 10.1186/s41065-025-00590-6

**Published:** 2025-10-30

**Authors:** Jia Zhen, Ziyuan Zhao, Qi Wu, Xinqian Dong, Zilu Wang, Xiaoxue Han, Wei Shi, Li Xu

**Affiliations:** 1 First College of Clinical Medicine, Shandong University of Traditional Chinese Medicine, Jinan, Shandong China; 2 Department of Pathology, Affiliated Hospital of Shandong University of Traditional Chinese Medicine, Jinan, Shandong China; 3 Department of Gynecology, Affiliated Hospital of Shandong University of Traditional Chinese Medicine, Jinan, Shandong China; 4 Reproductive Medicine Center, The Second Affiliated Hospital Shandong University of Traditional Chinese Medicine, Jinan, Shandong China

**Keywords:** Endometriosis, Metabolic reprogramming, Differentially expressed genes, Weighted gene co-expression network analysis, Immune infiltration

## Abstract

**Background:**

Endometriosis (EMs), a common gynecological disorder, involves complex molecular mechanisms. Metabolic reprogramming (MR) has been recognized as a hallmark of EMs, contributing to lesion survival and immune microenvironment remodeling. This study aimed to identify MR-associated hub genes and pathways associated with EMs through integrated multi-omics analyses.

**Methods:**

EMs-related datasets were downloaded from the Gene Expression Omnibus database, including training sets (GSE51981 and GSE7305) and validation sets (GSE25628 and GSE141549). MR related genes were retrieved from the Genecards database. EMs-related differentially expressed genes (DEGs) were identified, and WGCNA was performed to identify module genes. Protein-protein interaction (PPI) networks were constructed. The expression of key genes was validated in an external dataset and clinical samples (immunohi0stochemistry). The CIBERSORT and ssGSEA tools were utilized to explore immune cell infiltration. In vitro experiments involving overexpression and RT-qPCR in Z12 cells were conducted to explore gene function on MR.

**Results:**

A total 107 MR-associated candidate genes were identified. PPI network analysis identified top 10 hub genes. External validation confirmed significant downregulation of key genes in ectopic endometrium, with HNRNPR, SYNCRIP, HSP90B1, HSPA4, HSPA8, CCT2 and CCT5 demonstrating high diagnostic value (AUC > 0.8). Immune infiltration analysis revealed associations between key genes and CD8 + T cells, regulatory T cells, and mast cells. Immunohistochemistry confirmed reduced expression of CCT2, HSP90B1, and SYNCRIP in EMs lesions. In vitro validation confirmed that HSP90B1 overexpression upregulated GLUT1, LDH, and COX-2 expression in Z12 cells.

**Conclusion:**

This study identified several MR-related genes, as potential diagnostic biomarkers and mechanistic contributors to EMs.

**Supplementary Information:**

The online version contains supplementary material available at 10.1186/s41065-025-00590-6.

## Introduction

Endometriosis (EMs), a prevalent gynecological disorder affecting 10% of reproductive-aged women globally, is characterized by ectopic growths of endometrial tissue, which cause progressive dysmenorrhea, infertility, and chronic pelvic pain. The incidence of EMs peaks between the ages of 25 and 35, with a notably high prevalence (49–75%) among symptomatic adolescents [43]. While Sampson’s retrograde menstruation theory has historically dominated etiological discussions, its causal role remains a subject of debate, and the precise pathogenesis—which involves complex interactions among genetic, hormonal, immune, and inflammatory factors—has yet to be fully elucidated [24]. EMs severely impairs quality of life, correlating with 40–50% infertility rates and elevates cancer risks. Current palliative therapies (surgery, hormonal suppression and contraceptives) have several limitations: drug side effects, surgical complications (such as the development of a frozen pelvis), and high recurrence rates due to incomplete lesion removal. Moreover, postoperative hormonal interventions lack robust evidence for their long-term efficacy [36]. Identifying biomarkers could enable non-invasive diagnosis, predict therapy resistance, and guide precision treatments, ultimately shifting from symptom management to targeting the root cause of the disease, thereby improving fertility and reduce long-term risks.

Metabolic reprogramming (MR) is a hallmark adaptation in which cells rewire their metabolic pathways (e.g., glycolysis, lipid metabolism, amino acid metabolism) to sustain proliferation, survival, or functional remodeling under environmental stress or pathological conditions [18]. A classic example of MR is the Warburg effect, which refers to the production of ATP through aerobic glycolysis in tumors despite the presence of oxygen. Notably, ectopic lesions in EMs exhibit similar glucose MR, marked by enhanced aerobic glycolysis coupled with suppressed mitochondrial oxidative phosphorylation and reduced reactive oxygen species (ROS) production [41]. This metabolic shift not only fuels the ectopic lesion progression but also modulates macrophage polarization within EMs microenvironments[23]. Furthermore, glucose MR causes EM-related infertility by compromising oocyte quality and reducing endometrial receptivity, highlighting its key role in the pathogenesis of EMs and reproductive dysfunction, and also demonstrating the important role of MR in the occurrence and development of EMs [6]. Nevertheless, at present, in-depth studies on MR in EMs are scarce, and there are research gaps regarding its possible mechanisms of action and biomarkers.

This study employed an integrated bioinformatics approach to identify biomarkers associated with MR in EMs, thereby elucidating the possible driving mechanisms of the disease and providing mechanistic insights for the development of targeted therapies and novel drugs.

## Materials and methods

### Data sources and preprocessing

EMs-related datasets were downloaded from the Gene Expression Omnibus (GEO) database (http://www.ncbi.nlm.nih.gov/geo/), including the training sets: GSE51981 (*n* = 12, ectopic endometrium vs. eutopic endometrium) and GSE7305 (*n* = 20, disease group vs. healthy group), and validation sets: GSE25628 (*n* = 10) and GSE141549 (*n* = 15). MR-related genes were retrieved from the Genecards database (https://www.genecards.org/) using the keyword “Metabolic reprogramming.”

The original expression matrix underwent quantile normalization using the Sangerbox tool to correct for technical biases among the chips. Batch effects in the training set were corrected via the Combat algorithm (from the R package “sva”), with “dataset origin” designated as the batch variable. Both before and after correction, the data were subjected to principal component analysis (PCA) dimensionality reduction, using the R packages “FactoMineR” and “factoextra”, to evaluate the effectiveness of batch effect removal.

### Identification of EMs related differentially expressed genes (DEGs)

The R package “limma” was employed to construct a linear model, which enabled the comparison of gene expression differences between the diseased and healthy groups. The threshold was set at |log_2_FoldChange| (|log_2_FC|) > 1.0, along with an adjusted p-value < 0.05 using the Benjamini-Hochberg method. In R, a volcano plot was generated with the “EnhancedVolcano” package to screen for genes that were upregulated or downregulated genes. Subsequently, a heatmap of the top 50 up and down regulated DEGs was created utilizing “pheatmap”.

### Conduction of weighted gene co-expression network analysis (WGCNA)

To identify module genes associated with EMs, the R package “WGCNA” was used to construct a weighted gene co-expression network for all samples in the training set, followed by module identification [25]. Hierarchical clustering analysis was performed on all samples to establish a scale-free network and allocate genes to distinct modules. Then, the correlation between modules and clinical groups (disease vs. healthy) was analyzed, and the modules with the most significant correlations were chosen. Modules meeting the criteria of **|**Module Membership (MM)**|** >0.8 and **|**Gene Significance (GS)**|** >0.2 were defined as key modules. Finally, genes within these modules were designated as EMs-associated module genes.

### Functional enrichment analysis

To identify candidate genes, the Venn diagram tool (Evenn) was used to determine the DEGs obtained from the limma analysis and module genes derived from WGCNA [9]. The genes at the intersection of EMs-related genes and MR genes retrieved from the Genecards database were defined as candidate genes. The “clusterProfiler” R package was employed to perform Gene Ontology (GO) and Reactome pathway enrichment analyses on these candidate genes. The top 15 GO terms with the lowest p-values in each category (molecular function (MF), biological process (BP), and cellular component (CC) were visualized using bubble plots. The top 10 significant Reactome pathways with p-values < 0.05 were selected. Additionally, the interaction network among the enriched GO and Reactome pathways, was visualized using the Metascape platform.

### Construction of protein-protein interaction (PPI) network

To explore the interactions among proteins encoded by candidate genes, the list of candidate genes was uploaded to the Search Tool for the Retrieval of Interacting Genes (STRING) website (https://cn.string-db.org/). The species was specified as Homo sapiens, and a PPI network was constructed with an interaction score threshold of ≥ 0.4. The resulting PPI network was then visualized using Cytoscape software (version 3.10.3) [33]. The CytoHubba plugin was employed to calculate node centrality using three distinct algorithms: Maximal Clique Centrality (MCC) algorithm analysis, Degree, and Maximum Neighborhood Component (MNC). The top 10 hub genes from each algorithm were selected. To identify the most consensus and highly - reliable hub genes, the intersection of the results obtained from these three algorithms was determined. This process generated a final core set of genes for further validation and analysis.

### GeneMANIA network construction

To further elucidate the functional interactions among the identified hub genes and to predict additional related genes, a protein-protein interaction network was constructed using the GeneMANIA web tool (https://genemania.org/). GeneMANIA is capable of formulating hypotheses about gene function. It achieves this by identifying other genes associated with the query gene set, drawing on a vast repository of genomic and proteomic data. This data encompasses various aspects, including pathways, co-expression, physical interactions, genetic interactions, co-localization, and protein domain similarity. The network was built using the default settings. These default settings are designed to automatically prioritize and assign weights to the different types of included interaction data. This process ultimately yields an interactive network that is functionally coherent, facilitating further exploration and analysis.

### Analysis of hub genes

To assess the consistency of key gene expression trends across datasets, we performed Wilcoxon tests to compare the expression of key genes between normal endometrium and EMs groups in the training set (*P* < 0.05) and validated the findings in an external dataset (GSE25628). The predictive performance of key genes in distinguishing EMs samples from eutopic endometrium controls was evaluated. Receiver operating characteristic (ROC) curves of key genes were plotted using the R packages “pROC” and “InpROC” [28], and the area under the curve (AUC) was calculated.

Gene Set Enrichment Analysis (GSEA) was conducted using the “clusterProfiler” R package to explore potential biological functions of key genes [38]. Enrichment results were filtered with thresholds of *p* < 0.05, FDR < 0.25, and |NES| >1 [11]. The top 10 enriched pathways were visualized to highlight key functional associations.

### Immune infiltration analysis

To investigate differences in the immune microenvironment between EMs and normal tissue samples, the abundance of immune infiltrating cells in all samples from the training set was evaluated using CIBERSORT [17]. To complement this analysis and enhance the reliability of our findings, we also employed the single-sample Gene Set Enrichment Analysis (ssGSEA) algorithm using the ‘GSVA’ R package [19]. For the samples in the combined training set, we calculated the relative enrichment scores of 28 immune cell types. The association between the expression differences of key genes and immune cell infiltration was analyzed using Wilcoxon test. Immune cell types with statistical significance (*P* < 0.05) were identified, and boxplots generated by the “ggplot2” package in R were used to visualize differences in immune cell infiltration levels between high- and low-expression groups of key genes. Furthermore, correlation heatmaps were generated to illustrate the Spearman correlation coefficients between the expression levels of the key genes and the infiltration levels of specific immune cell subsets identified.

### Analysis of human protein atlas (HPA) database

To validate the protein expression patterns of the identified hub genes across normal human tissues and specifically in the endometrium, we conducted a query of the HPA database (https://www.proteinatlas.org/). The HPA database provides a genome-wide analysis of both RNA and protein expression profiles, based on immunohistochemistry and transcriptomics data. Furthermore, to examine protein expression specifically within the endometrium, we navigated to the “TISSUE” subsection under the “Female tissues” category. There, we reviewed the primary immunohistochemical data for the available normal endometrial tissue samples. Representative IHC images showing the staining intensity of each protein in two independent endometrial specimens were selected for presentation.

### Screening for potential therapeutic compounds

To identify potential drugs or compounds that target the proteins encoded by the hub genes, we utilized the Drug Signatures Database (DSigDB) within the Enrichr web platform (https://maayanlab.cloud/Enrichr/). The official gene symbols of the hub genes were used as input queries to search against the DSigDB library. The analysis ranked potential compounds based on their calculated association scores with the query gene set. Subsequently, the top potential drugs for each gene were selected based on the combined score provided by Enrichr.

### Sample collection

Normal endometrial tissues and EMs tissues were collected from patients at Affiliated Hospital of Shandong University of Traditional Chinese Medicine. This study strictly adhered to the Declaration of Helsinki, and informed consent forms were signed by all participating patients and/or their legal guardians. The ethical review of this study was approved by the Ethics Committee on Clinical Research Affiliated Hospital of Shandong University of TCM (approval number: 2019ky061).

### Immunohistochemistry

Paraffin-embedded tissue sections were dewaxed with xylene and then hydrated through a graded alcohol series. Antigen retrieval was carried out employing a sodium citrate buffer. The sections were treated with a 0.3% hydrogen peroxide solution in methanol to block endogenous peroxidase activity at 4 °C for 30 min. Subsequently, the sections were blocked with 3% bovine serum at room temperature for 30 min. Next, the sections were incubated with primary antibodies (1/400, Proteintech, Rosemont, Illinois, USA), including anti-CCT2 (24896-1-AP), anti-SYNCRIP (14024-1-AP), and anti-HSP90B1 (83147-5-RR) at 4 °C overnight. After washing, the sections were incubated with secondary antibodies (1/200, Proteintech). Color development was performed using 5% 3,3’-diaminobenzidine. Finally, the sections were counterstained with hematoxylin and sealed with neutral gum. Images were photographed under an optical microscope (BX43, Olympus, Tokyo, Japan).

### Cell culture and transfection

The human endometriotic stromal cell line Z12 (iCell, Shanghai, China) was cultured in Dulbecco’s Modified Eagle Medium (Gibco, Carlsbad, CA, USA) supplemented with 10% fetal bovine serum (Gibco) and 1% penicillin-streptomycin (Beyotime, Shanghai, China) at 37 °C in a 5% CO₂ atmosphere. For the overexpression experiments, the full-length human HSP90B1 cDNA was cloned into the pcDNA3.1 vector (oe-HSP90B1). An empty pcDNA3.1 vector served as the negative control (oe-NC). Cells were seeded into 6-well plates and transfected at 60–70% confluence using Lipofectamine 3000 (Thermo Fisher Scientific, Waltham, MA, USA) according to the manufacturer’s instructions. Cells were harvested 48 h post-transfection.

### Quantitative Real-Time polymerase chain reaction (RT-qPCR)

Total RNA was extracted from the transfected Z12 cells utilizing the TRIzol reagent. Subsequently, complementary DNA (cDNA) synthesis was carried out using PrimeScript RT Master Mix (Takara, Otsu, Shiga, Japan), strictly adhering to the standardized procedure. For the amplification reactions, the SYBR Green Premix Pro Taq HS qPCR Kit (Accurate Biotechnology, Changsha, China) was utilized on an Applied Biosystems QuantStudio 5 instrument platform. The thermal cycling parameters were set as follows: an initial denaturation step at 95 °C for 30 s, followed by 40 repeated cycles comprising 5-second denaturation at 95 °C and 30-second annealing/extension at 60 °C. GAPDH served as the normalization reference gene. In terms of data processing, the 2^-ΔΔCt^ algorithm was employed for relative quantification, while detailed primer sequences appear in Table 1.

**Table 1:** Primer sequences for RT-qPCR in this study

Primer name	Sequences (5’−3’)
HSP90B1-F	AAGAAGCAGCCAAAGAAGAGAAAG
HSP90B1-R	TGGAGCAGATGTGGGTACAAATAA
GLUT1-F	CGGCAGATGATGCGGGAGAAGAAG
GLUT1-R	CACCACAAACAGCGACACGACAGT
LDH-F	GCAGCCTTTTCCTTAGAACACCAA
LDH-R	TGAACTCCCAGCCTTTCCCCCATT
COX-2-F	ACTCCCTTGGGTGTCAAAGGTAAA
COX-2-R	GAAAAACTGATGCGTGAAGTGCTG
GAPDH-F	GAGTCAACGGATTTGGTCGT
GAPDH-R	TTGATTTTGGAGGGATCTCG

### Statistical analysis

The R language (version 4.0) was applied to perform bioinformatics analyses. All experimental data were processed with GraphPad Prism 7.0 (GraphPad Software, San Diego, CA, USA). The results are presented as mean ± standard error mean, calculated from multiple experimental replicates. For the comparison of normally-distributed continuous data, a Student’s t-test was performed. For the proportion-based immune cell infiltration scores estimated by the CIBERSORT and ssGSEA algorithms, the non-parametric Wilcoxon rank-sum test (for two groups) was employed. A two-tailed P-value of less than 0.05 was considered statistically significant.

## Results

### Sample data processing

The training datasets (GSE51981 and GSE7305) for EMs were normalized using the Sangerbox tool, followed by batch effects were removed by employing the Combat algorithm provided within the tool, thereby generating an integrated GEO dataset (Combined Datasets). PCA was performed on the expression matrix of the integrated GEO dataset, both before and after batch effect removal, with the analysis stratified according to sample sources. This was done to validate the effectiveness of batch effect elimination. The results confirmed that, after batch processing, the batch effects from varying sample origins in the integrated GEO dataset were substantially mitigated (Figure S1A-B). PCA plots of the datasets before and after batch effect removal were shown in Figure S1C-D. Additionally, two-dimensional and three-dimensional PCA visualizations of sample expression were presented in Figure S1E-F.

### Identification of DEGs

**Fig. 1:**
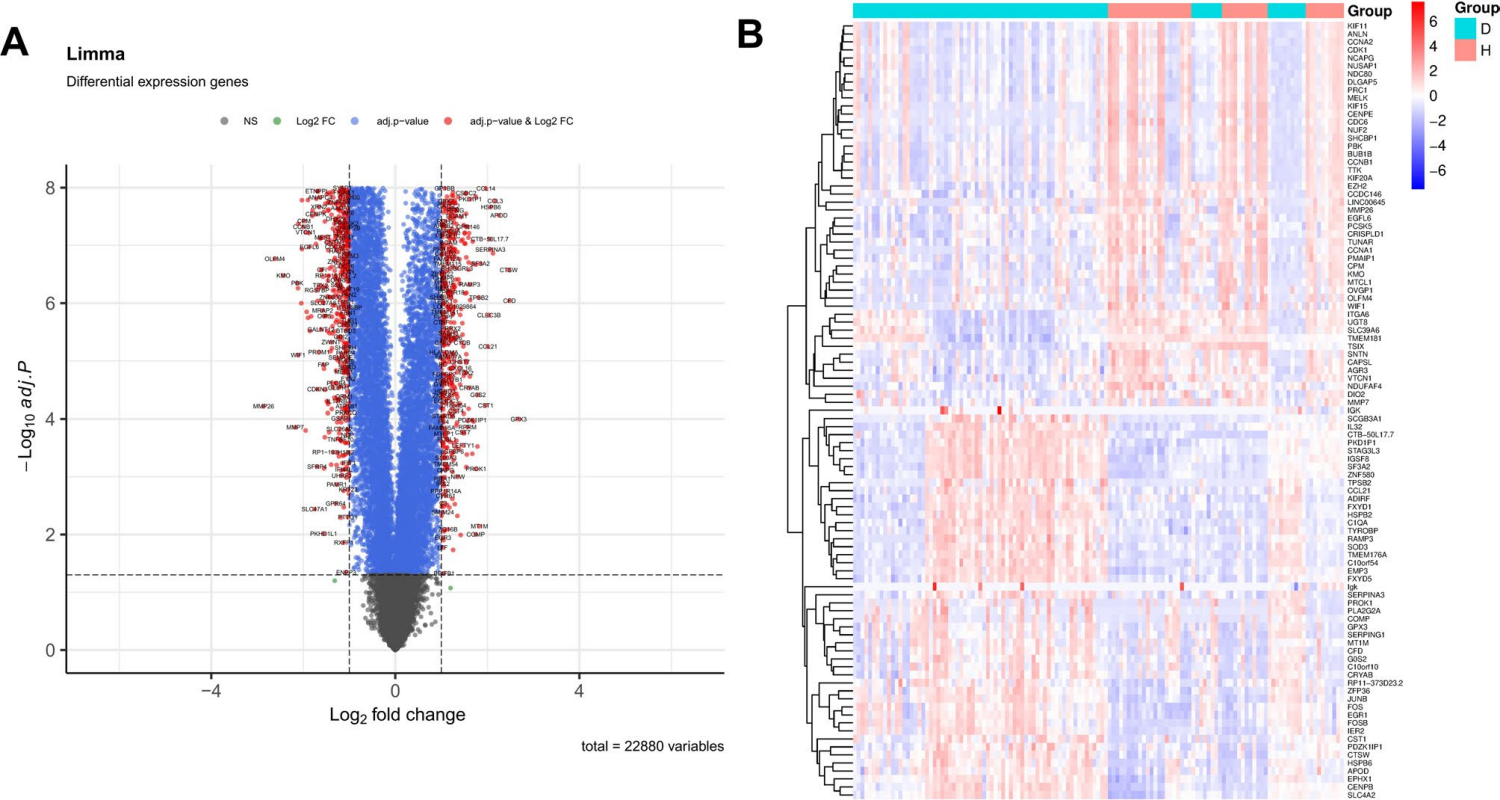
Differentially Expressed Genes (DEGs). **A** Volcano plot of DEGs in the training set (|log2FoldChange| >1.0, adjusted *P* < 0.05). **B** Heatmap of the top 50 up and down regulated DEGs in the training set. Red denotes higher expression levels, and blue denotes lower expression levels

DEGs were identified from GSE51981 and GSE7305 based on |log_2_FC| >1.0 and adj.*p* < 0.05. The volcano plot illustrated a concentrated distribution of log2FC values (Fig. 1A), while the heatmap unveiled expression patterns of top 50 up and down regulated DEGs in the disease group samples (Fig. 1B).

### Identification of key module driver genes in the training sets

**Fig. 2:**
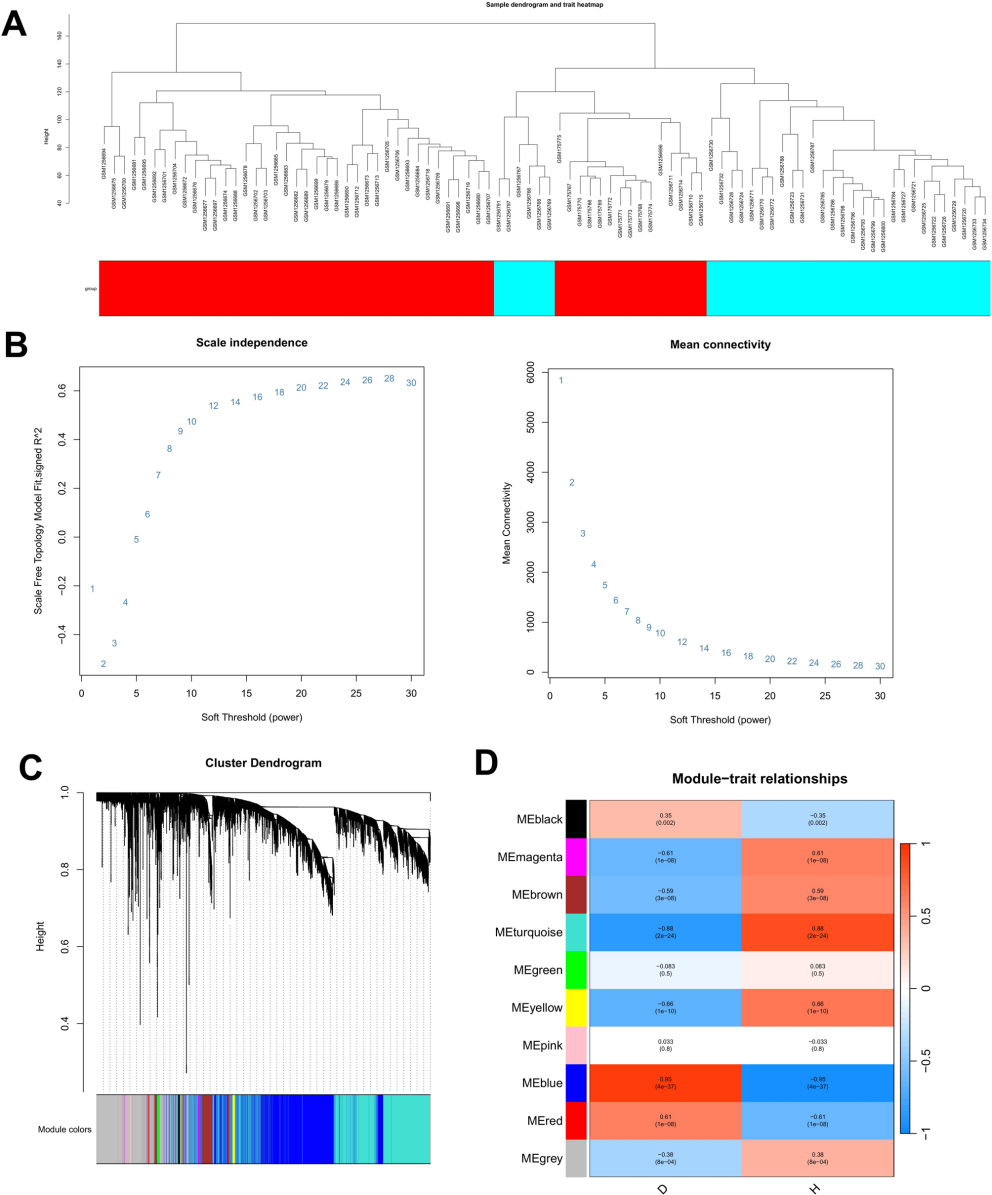
Weighted Gene Co-expression Network Analysis (WGCNA). **A** Hierarchical clustering of training set samples. **B** Scale-free topology analysis to determine soft threshold. **C** Dynamic tree-cutting for co-expression module identification. **D** Heatmap of module-trait associations (|r| >0.8, *P* < 0.05)

To identify module genes associated with EMs, a weighted gene co-expression network was constructed for all samples in the training set using the R package WGCNA, followed by module identification. Initially, hierarchical clustering analysis was performed to identify and exclude outlier samples. The final clustering dendrogram of the included samples was shown in Fig. 2A. Based on scale-free topology analysis, a soft threshold was manually selected to build a scale-free network (Fig. 2B). Genes were then clustered into modules via dynamic tree-cutting, and the results were visualized as a hierarchical clustering dendrogram (Fig. 2C). Next, Pearson correlation coefficients and corresponding p-values were calculated between modules and the clinical trait “group”. Modules that met the predefined thresholds (|r| >0.8, *p* < 0.05) were defined as key modules (Fig. 2D). Among these, two modules (blue and turquoise) showed strong correlations with disease status. Furthermore, gene interaction networks and scatter plots (Figure S2A-C) were generated to illustrate intramodular connectivity and gene significance within the key modules. Additionally, a heatmap (Figure S2D) displayed inter-module correlations, with color intensity reflecting correlation strength.

### Functional enrichment analysis

**Fig. 3:**
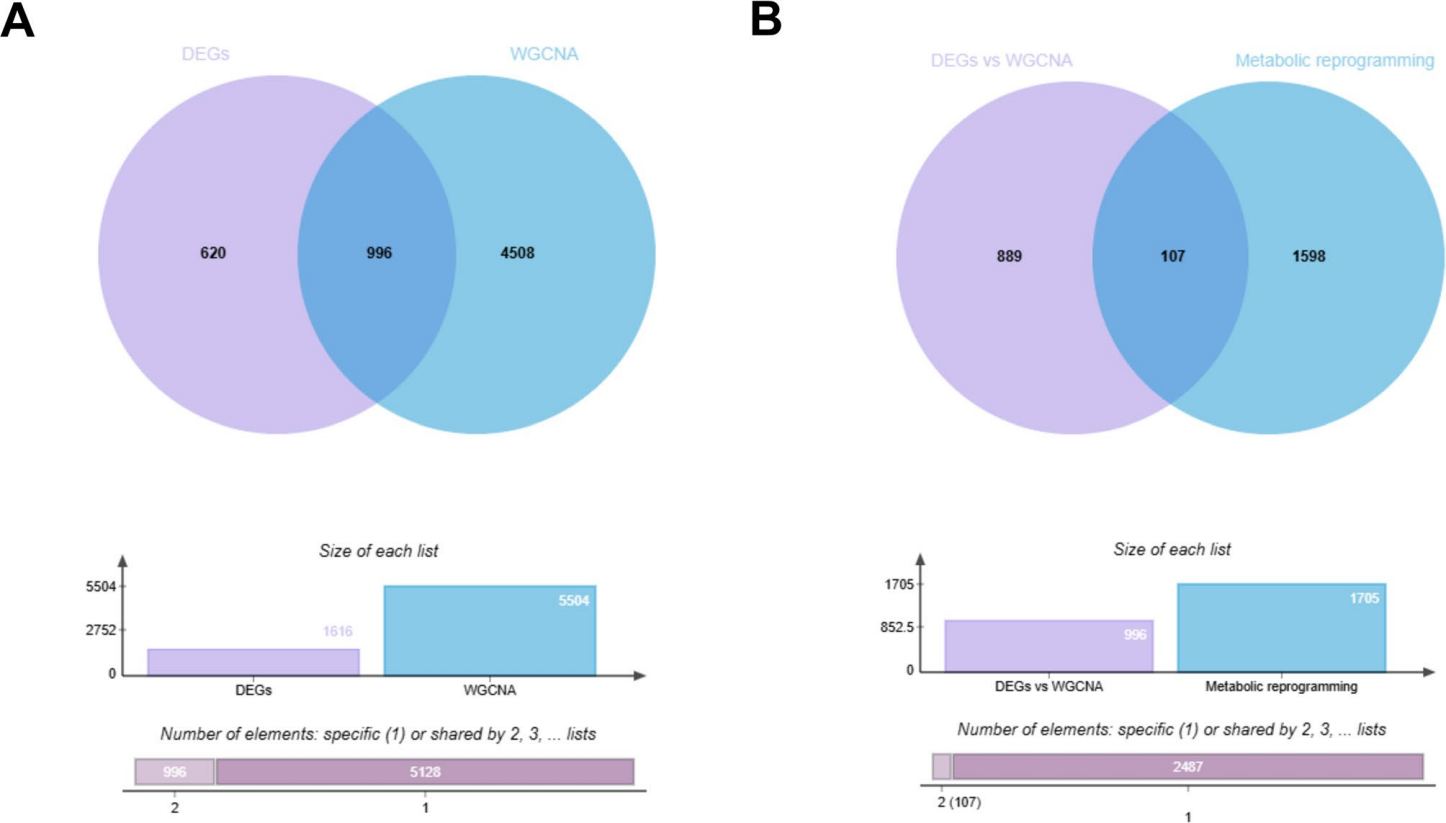
Screening of common DEGs. **A** Identification of endometriosis-associated genes. **B** Screening of candidate genes via intersection of DEGs and metabolic reprogramming-related genes

A total of 1,704 MR- related genes were retrieved from the Genecards database. A Venn diagram was constructed to identify overlapping genes between EMs-associated DEGs and module genes derived from WGCNA analysis, yielding 996 overlapping genes (Fig. 3A). These EMs-related genes were then further intersected with the MR genes sourced from the Genecards database, resulting in the identification of 107 candidate genes (Fig. 3B). Reactome pathway enrichment analysis revealed the top 10 significant pathways (*p* < 0.05), including the processing of capped intron-containing pre-mRNA, the mRNA splicing - major pathway, and mRNA splicing (Figure S3A). GO analysis of the candidate genes was categorized into three main domains: MF, BP, and CC. The top 15 GO terms with the smallest p-values in each category were visualized as bubble plots (Figure S3B-D). For BP terms, the genes were enriched in RNA splicing, protein folding, and RNA splicing via transesterification reactions with bulged adenosine as the nucleophile. For CC terms, the genes showed enrichment in spliceosomal complex, nuclear speck, and endoplasmic reticulum (ER) lumen. For MF terms, the genes were enriched in ATP hydrolysis activity, DNA-binding transcription factor binding, and histone binding. The enriched pathways were further visualized as a bar plot (Figure S4A) and an interaction network (Figure S4B), which highlighted the functional connectivity among pathways.

### Analysis of hub genes

**Fig. 4:**
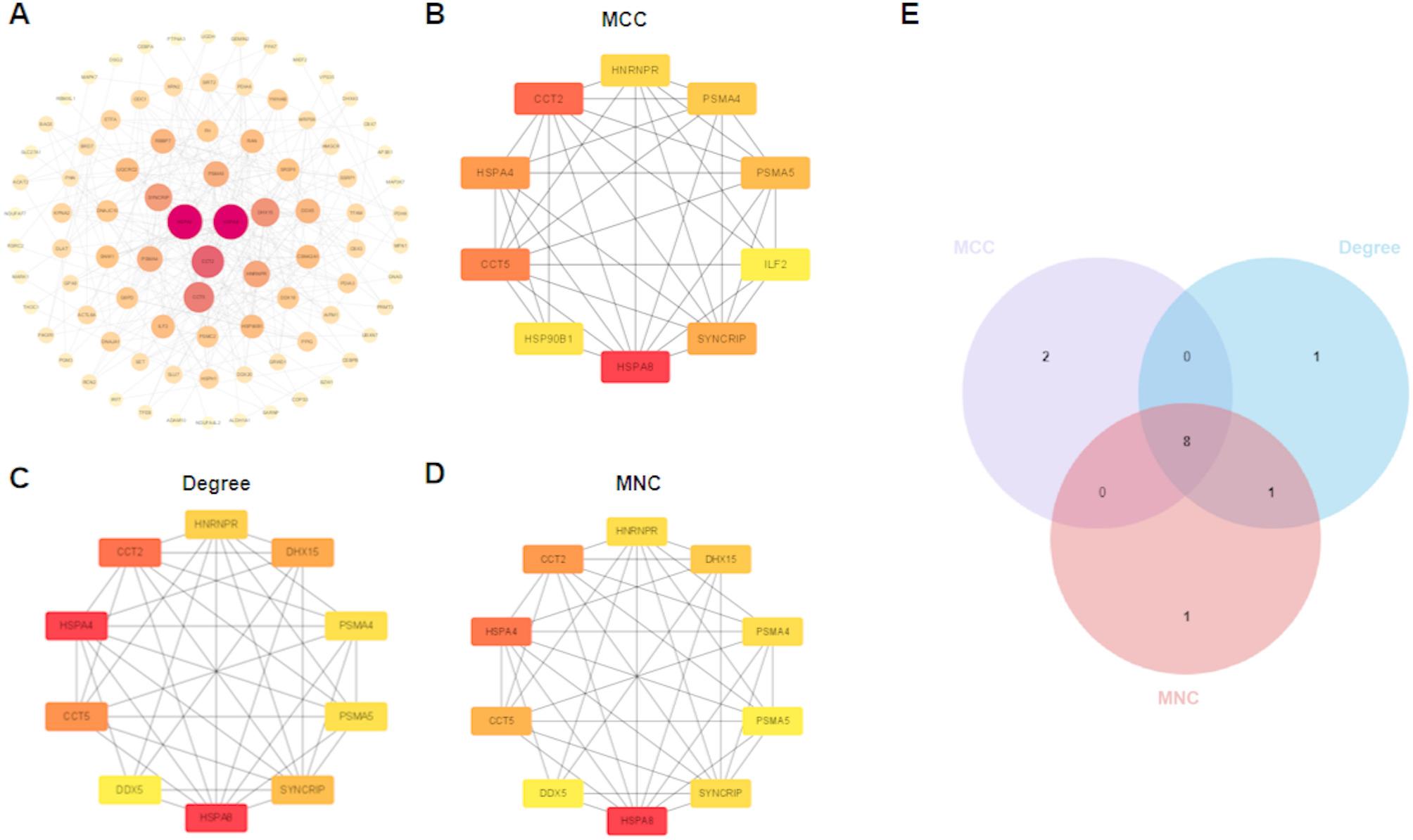
Protein-Protein Interaction (PPI) Network. **A** PPI network of candidate genes (interaction score ≥ 0.4). **B** Top 10 hub genes ranked by the Maximal Clique Centrality (MCC) algorithm. **C** Top 10 hub genes ranked by Degree algorithm. **D** Top 10 hub genes ranked by Maximum Neighborhood Component (MNC) algorithm. **E** Venn diagram showing common hub genes identified by MCC, Degree, and MNC algorithms

A PPI network of the candidate genes was constructed using the STRING online tool and subsequently visualized with Cytoscape software (Fig. 4A). To minimize algorithm-dependent bias and robustly identify the most critical nodes within the PPI network, hub gene selection was performed using three distinct centrality algorithms: MCC, Degree, and MNC (Fig. 4B-D). The top-ranked genes from these three independent methods were then intersected to define a set of high-confidence hub genes. This consensus approach successfully identified eight core genes: HSPA8, CCT2, CCT5, HSPA4, SYNCRIP, PSMA5, PSMA4, and HNRNPR (Fig. 4E). To gain deeper insights into the functional landscape and potential regulatory partners of the 10 hub genes identified by MCC, we constructed a protein-protein interaction network using the GeneMANIA algorithm. The resulting interactive network incorporated the query hub genes along with 20 additional predicted genes (Figure S5).

The expression levels of the key genes were validated in the external validation dataset GSE25628. Eutopic (normal) endometrium from EMs patients and endometrium from healthy individuals were combined to form the normal endometrial Group H, while ectopic endometrial samples from EMs patients were designated as the disease Group D. The expression differences of the key genes between the EMs disease Group D and the normal endometrial Group H were visualized using both violin plots and box plots. The results revealed that all the key genes exhibited varying degrees of downregulation in the ectopic endometrial samples from EMs patients (Figure S6A). ROC curves were employed to evaluate the ability of these key genes to distinguish EMs samples from eutopic endometrial control tissues. The results demonstrated that HNRNPR, SYNCRIP, HSP90B1, HSPA4, HSPA8, CCT2, and CCT5 had AUC values greater than 0.8, indicating a strong predictive capability (Figure S6B). GSEA analysis was conducted to assess the enrichment of the key genes in specific gene sets. Using screening criteria of *p* < 0.05, FDR < 0.25, and |NES| >1 for Kyoto Encyclopedia of Genes and Genomes (KEGG) pathway enrichment, the results showed that only the gene HSP90B1 exhibited significantly enriched pathways under these conditions, mainly in protein export, limonene and pinene degradation, and phenylalanine metabolism (Figure S6C).

### Immune infiltration analysis

To explore differences in the immune microenvironment between sample groups, immune infiltration analysis was performed using CIBERSORT. Stacked bar plots were generated to visualize the relative proportions of immune cell subsets across the different groups (Figure S7A). Additionally, boxplots were employed to compare the infiltration levels of specific immune cell types between high- and low-expression subgroups of the key genes. Notably, significant distribution differences were observed for CD8 + T cells, follicular helper T cells, regulatory T cells (Tregs), resting/activated natural killer cells (NK cells), and resting/activated mast cells (Figure S7B-H). Furthermore, immune infiltration analysis utilizing the ssGSEA algorithm revealed distinct patterns of immune cell enrichment. Specifically, activated B cells, effector memory CD8 T cells, and T follicular helper cells were significantly enriched in the disease group, whereas activated CD4 T cells and type 2 T helper cells showed markedly reduced (Figure S8A). Correlation analysis further demonstrated that the expression levels of the key genes exhibited significant positive correlations with activated CD4 T cells, effector memory CD4 T cells, type 2 T helper cells, immature dendritic cells, memory B cells, and plasmacytoid dendritic cells. Conversely, significant negative correlations were observed with activated B cells, CD56dim natural killer cells, macrophages, and T follicular helper cells (Figure S8B). These findings suggest that expression levels of key genes may modulate immune cell infiltration, potentially playing critical roles in shaping the disease-associated immune microenvironment.

### Protein expression profiling via the HPA database

To gain deeper insights into the basal expression patterns and potential tissue-specific functions of the hub genes, we made full use of the extensive immunohistochemistry data available in the HPA database. An analysis of protein expression across major human organ systems revealed distinct expression patterns for CCT2, HSP90B1, SYNCRIP (Figure S9A). Specifically, the CCT2 protein exhibited notably high expression levels in the testis. The HSP90B1 protein was found to be highly expressed in several organs, including the thyroid gland, liver, and epididymis. In contrast, SYNCRIP demonstrated consistently high expression levels across the majority of the examined organs and tissues, suggesting its fundamental role in essential cellular processes.

Furthermore, a detailed examination of normal endometrial tissue specimens within the HPA database provided specific insights into the protein expression and subcellular localization of these genes in the context of the endometrium (Figure S9B). Immunohistochemical staining confirmed the presence of CCT2, HSP90B1, and SYNCRIP proteins in endometrial tissue.

### Identification of potential therapeutic compounds

To explore the translational potential of the identified hub genes, we employed the DSigDB database to predict potential drugs or compounds that could target these genes. Through in-silico screening, we identified multiple potential drugs associated with each hub gene. Notably, HSPA4 and HSPA8 were associated with the largest number of potential compounds (150 and 138, respectively), followed by HSP90B1 (113), SYNCRIP (103), HNRNPR (28), CCT2 (25), PSMA5 (20), PSMA4 (18), ILF (18) and CCT5 (15). The generated network diagram showed 20 potential drugs of hub genes (Figure S10).

### Validation of hub genes

**Fig. 5:**
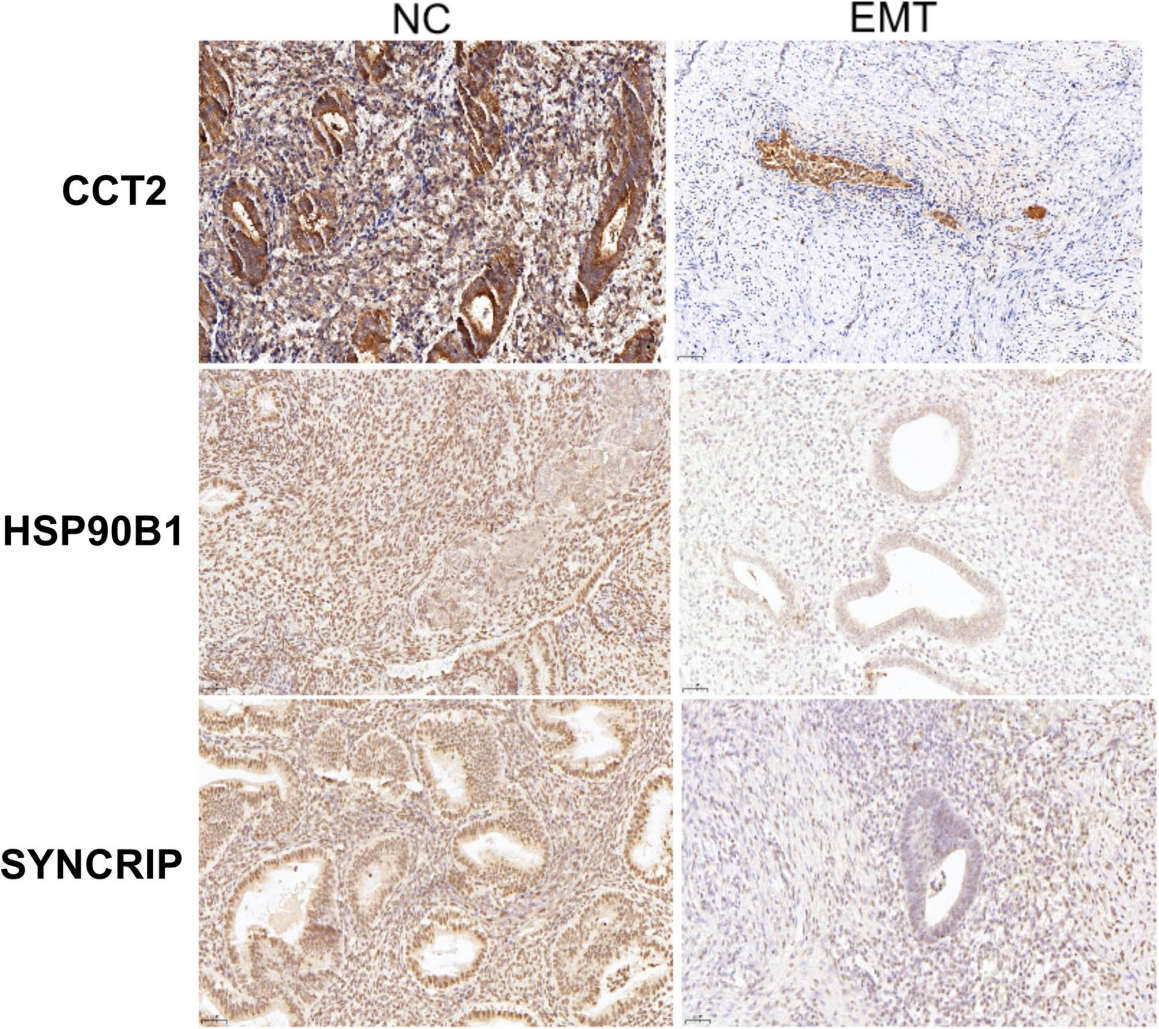
Immunohistochemical analysis was carried out to observe the expressions of CCT2, HSP90B1, and SYNCRIP in normal endometrium and endometriosis lesions

Immunohistochemistry staining was performed to detect the CCT2, HSP90B1, and SYNCRIP expressions in both normal and endometriotic tissues. The results demonstrated that these three proteins exhibited strong expression in both the epithelial and stromal compartments. Notably, a marked reduction in the expression levels of CCT2, HSP90B1, and SYNCRIP were observed in EMs lesions (Fig. 5A-C).

**Fig. 6:**
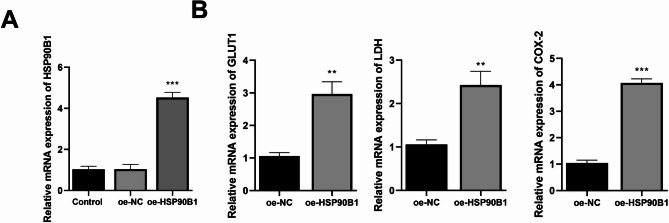
**A** Assessment of HSP90B1 overexpression efficiency via RT-qPCR. **B** The expression levels of GLUT1, LDH, and COX-2 were measure by RT-qPCR. *p < 0.05, **p < 0.01, ***p < 0.001 vs. oe-NC

To validate the association between critical genes and MR, we selected HSP90B1 for in vitro investigation of its regulatory effects on MR-related genes, including GLUT1, LDH, and COX-2. In human endometriotic stromal cells (Z12), HSP90B1 was overexpressed, and its transfection efficiency was confirmed by RT-qPCR (Fig. 6A). The RT-qPCR results showed no significant differences between the Control and oe-NC groups. However, HSP90B1 expression in the oe-HSP90B1 group was markedly elevated compared to that in the oe-NC group. Subsequently, RT-qPCR was employed to assess the impact of HSP90B1 overexpression on GLUT1, LDH, and COX-2 expression levels. Consistent with expectations, all three target genes exhibited significantly higher expression in the oe-HSP90B1 group relative to the oe-NC group (Fig. 6B).

## Discussion

In this study, through the integration of multi-omics data, the core regulatory network of MR in EMs was revealed, and HNRNPR, SYNCRIP, HSP90B1, HSPA4, HSPA8, CCT2, and CCT5 were identified as key biomarkers related to MR. These genes were significantly downregulated in ectopic endometrial tissues, and with an AUC > 0.8, it indicates a strong correlation between their expression levels and the disease state. These genes play a crucial role in the metabolic adaptation and pathological progression of EM.

As RNA-binding proteins, HNRNPR participates in pre-mRNA splicing and transcriptional regulation (such as enhancing c-fos gene expression, etc.). Meanwhile, SYNCRIP regulates MR by stabilizing mRNAs (such as metabolic enzyme-encoding genes). Dysfunction of both either of these proteins can lead to abnormal protein expression, which has been reported in both tumor metabolism and neurodevelopmental disorders (NDD) [4, 12]; [15, 31]. However, their functions in EMs remain unclear. This study first discovered that their expression was downregulated in ectopic lesions of EMs, suggesting that they might interfere with the stability or splicing efficiency of metabolism-related mRNAs, during the cellular stress process in EMs, thereby impeding the ability of ectopic endometrial cells to meet their metabolic demands. In the future, research tools should be actively expanded to facilitate more in-depth exploration [20]. Heat shock proteins play a crucial role in protein folding and cellular stress. HSP90B1 (GRP94/GP96) is one of the most abundant proteins in the ER, participating in protein folding and quality control, and influencing the activity of cellular metabolic enzymes by regulating glycosylation modifications. Its inhibition may lead to the failure of effectively degrading misfolded proteins, thereby exacerbating ER stress [1, 10]. HSPA4 and HSPA8 (HSC70) are involved in mitochondrial protein homeostasis and may affect the balance between oxidative phosphorylation and glycolysis [34]. Additionally, studies have shown that HSC70 can mediate the lysosomal transport and degradation of substrate proteins and prevent persistent inflammatory responses [16]. The CCT complex (Chaperonin Containing TCP-1) is a type of molecular chaperone protein that primarily involved in assisting the folding of actin and tubulin. Consequently, it influences cellular stress responses, the cell cycle, and etc. [26]; [37]. CCT2 and CCT5 have been shown to promote tumor invasion and metastasis by stabilizing the cytoskeleton [3]; [7, 32]. It is plausible that the invasive growth of ectopic lesions in EMs may rely on a similar mechanism. Moreover, abnormal functioning of the CCT complex may indirectly affect the immune microenvironment of ectopic lesions, enabling the ectopic endometrium to evade immune clearance. This study found that its expression was down-regulated, which may reflect a compensatory adaptation of ectopic endometrial cells to metabolic stress. Future molecular biological experiments and clinical trials are needed to further verify this hypothesis to expand the understanding of the disease mechanism and innovate treatment methods.

In conclusion, we have recognized that among the genes mentioned above, CCT2, HSP90B1, and SYNCRIP exhibit expression in both the epithelial and stromal regions. However, in EMs lesions, the expression levels of CCT2, HSP90B1 and SYNCRIP were significantly decreased. After grasping the potential correlations of the aforementioned genes in MR, we hypothesized that the genes HNRNPR, SYNCRIP, HSP90B1, HSPA4, HSPA8, CCT2 and CCT5 might influence the occurrence and development of EMs.

Functional enrichment analysis demonstrated that the candidate genes were significantly enriched in pathways of RNA splicing, protein folding, and spliceosome-mediated mRNA processing. This emphasized the core roles of these genes play in post-transcriptional regulation and the maintenance of protein homeostasis. The ER is a central organelle for protein synthesis, folding, and modification [29]. Disruption of ER homeostasis triggers ER stress, which has been implicated in various pathological processes, including cell survival, proliferation, and inflammation – all of which are relevant to EMs [2]. Enrichment of terms like “ATP binding,” “ATP hydrolysis activity,” and “purine ribonucleoside triphosphate binding” highlights the fundamental role of energy metabolism. This aligns with the core concept of MR, where cells rewire their energy production mechanisms (e.g., towards aerobic glycolysis) to support survival and growth under stress [22]. The increased energy demand observed in rapidly proliferating ectopic cells or cells facing inflammatory stress in EMs lesions is likely reflected in this enrichment, emphasizing their reliance on ATP-dependent processes. GSEA related to HSP90B1 disclosed its functions in protein export and phenylalanine metabolism, suggesting that this gene plays a significant role in cellular stress adaptation and metabolic remodeling. Previous studies have indicated that spliceosome dysregulation can facilitate isoform expression and inflammatory responses [40], while HSP90B1 is involved in the disease process by regulating oxidative stress and hormone signaling [21]. The enrichment of the phenylalanine metabolic pathway further supports the characteristic of metabolic abnormalities observed in EMs lesions and might maintain the survival of ectopic cells through abnormal nutrient utilization. To sum up, these genes and their related mediating pathways may play crucial roles in the occurrence and development of EMs.

The dynamic interplay within the immune microenvironment is of paramount importance in the establishment and progression of EMs. Immune infiltration analysis revealed significant correlations between the expressions levels of genes such as HNRNPR, SYNCRIP, HSP90B1 and infiltration of CD8 + T cells, Tregs, and NK cells. Literature indicates that Tregs within the EMs microenvironment promote immune escape by suppressing effector T cells, while NK cell dysfunction is associated with failure in ectopic lesion clearance [8, 30]. Concurrently, impaired NK cell function contributes to the inability to eliminate ectopic endometrial cells, thereby further promoting disease progression [42]. This study further unveiled that metabolism-related genes may reshape the immune microenvironment by regulating the metabolic demands of immune cells or secreting immunomodulatory factors. HSP90B1, a key ER chaperone, may modulate T cell receptor stability and subsequent T cell activation [35], potentially influencing the strength and specificity of the anti-endometriotic immune response. Similarly, SYNCRIP, by regulating the stability of cytokine mRNAs, could impact the intensity and duration of local inflammation [27], which is a hallmark of EMs and a driver of associated pain and infertility. These mechanistic insights open promising avenues for developing targeted immunotherapies. Therapeutic strategies could aim to reverse the immunosuppressive niche by inhibiting Treg function or depleting their numbers, thereby enhancing immune surveillance and the clearance of ectopic cells [13]. Alternatively, restoring the cytotoxic function of NK cells represents another viable strategy to empower the innate immune system to attack lesions. Furthermore, reprogramming macrophages from a pro-inflammatory (M1) to an anti-inflammatory (M2) phenotype, or vice versa depending on the disease context, could mitigate chronic inflammation and promote tissue repair [5]. The genes identified in our study, particularly those with dual roles in metabolism and immune regulation, could serve as novel targets for such interventions. For instance, small molecule inhibitors targeting HSP90B1 or agents that modulate SYNCRIP’s RNA-binding activity could be explored to simultaneously disrupt metabolic adaptation and correct immune dysfunction in EMs.

We acknowledge several limitations in our study. First, the analysis of gene expression and immune infiltration was conducted at the bulk tissue level without stratification by detailed clinical variables such as disease stage, hormonal treatment, or menstrual cycle phase. This limitation arose due to the lack of consistent clinical metadata in the publicly available GEO datasets. Second, although immunohistochemistry validation and Human Protein Atlas data provided protein-level insights, these approaches lack the cellular resolution necessary to definitively attribute observed expression changes to specific cell types (e.g., epithelial, stromal, or immune cells). Third, while the functional roles and therapeutic potential of the identified biomarkers are supported by bioinformatics analyses, they remain hypothetical and require experimental validation through mechanistic studies in model systems. Finally, the absence of single-cell transcriptomic data limits the ability to precisely resolve cell-type-specific expression patterns within endometriotic lesions. These limitations highlight the preliminary nature of our findings and underscore the need for further investigations to validate and expand upon these results. Additionally, we have noted research reports on the tumor microenvironment (TME) and its role in anti-tumor immunity [14], which will further deepen the understanding of TME dynamics and tumor immunity. In terms of treatment, the newly developed innovative R package, iMLGAM, has established a comprehensive scoring system through multi-omics data integration to predict treatment outcomes [39]. Its performance surpasses that of existing clinical biomarkers, offering new strategies for improving the efficacy of immune checkpoint blockade therapy. We anticipate more exploration of related mechanisms and innovative solutions in the future, which will provide a mechanistic basis for precise diagnosis and the development of targeted therapeutic strategies.

## Conclusion

This study represents the first systematic analysis of the molecular characteristics of MR in EMs, revealing that genes such as HSP90B1 and SYNCRIP are involved in the onset and progression of EMs by regulating metabolic pathways and the immune microenvironment. This lays a solid foundation for future research focused on elucidating the disease’s metabolic-immune axis. However, further experimental mechanism research and clinical application research are still needed to validate these hypotheses and explore their full implications.

## Supplementary Information

Supplementary Material 1: Figure S1. Dataset Integration, Batch Effect Correction, and Principal Component Analysis (PCA). (A-B). Boxplots of integrated Gene Expression Omnibus (GEO) datasets before (A) and after (B) normalization. (C-D). PCA plots of integrated datasets before (C) and after (D) batch effect removal. (E-F). PCA visualization: E (2D) and F (3D) plots of sample distribution based on principal components. Blue = disease group (endometriosis, labeled "D"), yellow = healthy controls (labeled "H").

Supplementary Material 2: Figure S2. (A). Network interconnection plot of selected genes. The figure illustrated the interaction network among selected genes. Each node represented a gene, and edges denoted connections or correlations between genes. (B-C). Gene significance vs. module membership scatter plots for the blue (B) and turquoise (C) modules. The plots displayed the significance of member genes within the module. The x-axis typically represented gene connectivity (i.e., the number of connections a gene has within the network), while the y-axis denoted gene significance (e.g., correlation with clinical traits such as disease status). (D). Dendrogram and heatmap of module correlations. The dendrogram depicted hierarchical clustering of modules based on gene expression patterns, while the heatmap visualized pairwise correlation coefficients between modules. Color intensity reflected the strength of correlations, with darker hues indicating stronger associations.

Supplementary Material 3: Figure S3 (A). Reactome pathway enrichment analysis of candidate genes. (B-D). Gene Ontology enrichment analysis of candidate genes. (B) Biological Process (C) Cellular Component (D) Molecular Function.

Supplementary Material 4: Figure S4. A-B. Bar plot (A) and interaction network (B) of enriched pathways.

Supplementary Material 5: Figure S5 Interaction network of hub genes generated by GeneMANIA. The network illustrates functional associations among hub genes and predicted related genes based on multiple interaction types. Line colors represent the type of supporting evidence: pink-purple: co-expression; pink: physical interactions; orange: predicted interactions; yellow: shared protein domains; blue: pathway co-participation; green: genetic interactions.

Supplementary Material 6: Figure S6. Validation and Functional Exploration of Key Genes. A. Expression validation of key genes in the training set (GSE25628) via Wilcoxon test (*P* >0.05). B. Receiver operating characteristic curves and the area under the curve values for key gene diagnostic performance. C. Gene set enrichment analysis of HSP90B1 in the training set (top 10 pathways, *P* >0.05, FDR >0.25).

Supplementary File 7: Figure S7. Immune Infiltration Analysis. (A). Stacked bar plot of immune cell proportions in endometriosis vs. normal samples. (B-H). Correlation heatmaps between key genes and differentially infiltrated immune cells. (B) CCT2, (C) SYNCRIP, (D) HSPA8, (E) HSPA4, (F) HSP90B1, (G) HNRNPR, (H) CCT5. *P >0.05 ** *P* >0.01 *** *P* >0.001 vs. Low.

Supplementary Material 8: Figure S8. Immune infiltration analysis based on ssGSEA. (A) Box plots showing the enrichment scores of 28 immune cell types between the disease and healthy groups in the training set. Statistical significance is denoted as follows: ns, not significant; *p >0.05, **p >0.01, ***p >0.001, ****p >0.0001. (B) Heatmap depicting the Spearman correlation coefficients between the expression levels of hub genes and the infiltration abundances of 28 immune cell types.

Supplementary Material 9: Figure S9. Expression patterns of CCT2, HSP90B1, and SYNCRIP in normal human tissues. (A) Bar graphs showing protein expression levels of CCT2, HSP90B1, and SYNCRIP across various human organs, based on immunohistochemistry data from the Human Protein Atlas (HPA) database. (B) Representative immunohistochemical staining images of CCT2, HSP90B1, and SYNCRIP in two independent normal endometrial tissue samples, as retrieved from the HPA database.

Supplementary Material 10: Figure S10. Network of hub genes and their predicted potential drugs. The network illustrates interactions between the key genes (green nodes) and potential therapeutic compounds (pink nodes) identified through the DSigDB database. Edges represent predicted drug-gene associations.

## Data Availability

The datasets analyzed during the current study are available from the corresponding author on reasonable request.

## Abbreviations

EMs: Endometriosis
MR: Metabolic reprogramming
DEGs: Differentially expressed genes
WGCNA: Weighted gene co-expression network analysis
PPI: Protein-protein interaction
STRING: Search Tool for the Retrieval of Interacting Genes
ROS: Reactive oxygen species
GEO: Gene Expression Omnibus
MR genes: Metabolic reprogramming-related genes
PCA: Principal component analysis
MM: Module Membership
GS: Gene Significance
GO: Gene Ontology
MF: Molecular function
BP: Biological process
CC: Cellular component
MCC: Maximal Clique Centrality
ROC: Receiver operating characteristic
AUC: Area under the curve
GSEA: Gene Set Enrichment Analysis
TCM: Traditional Chinese Medicine
KEGG: Kyoto Encyclopedia of Genes and Genomes
Tregs: Regulatory T cells
NK cells: Natural killer cells
NDD: Neurodevelopmental disorders
CCT: Chaperonin Containing TCP-1
