# Supplementary figures and images for: Identification of metabolic reprogramming-associated biomarkers in endometriosis through integrated bioinformatics analysis
Source: Hereditas. 2025 Oct 30;162:221. doi: 10.1186/s41065-025-00590-6 (PMC12574043; doi:10.1186/s41065-025-00590-6)

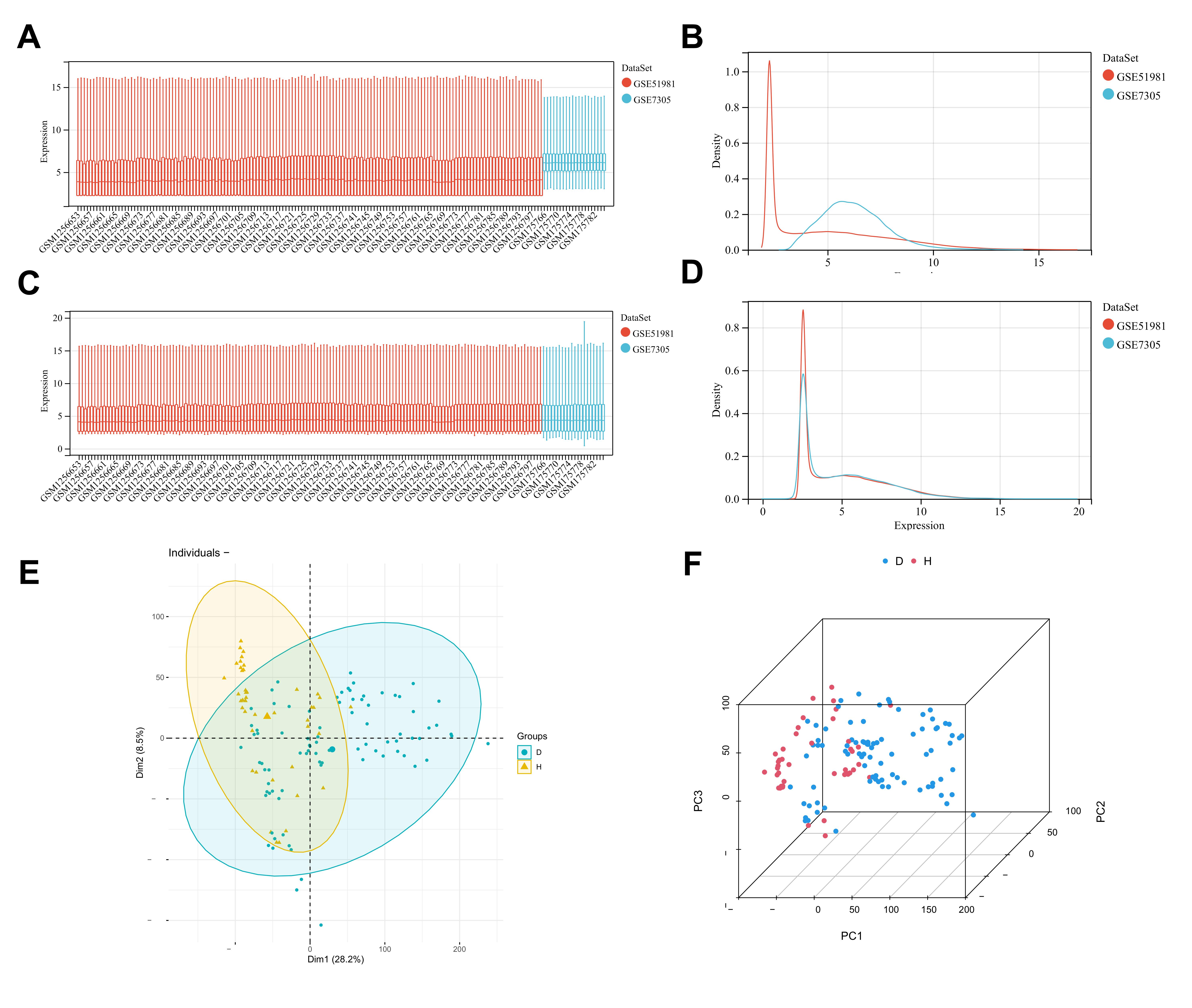

Supplement: Supplementary file 1 — Supplementary Material 1: Figure S1. Dataset Integration, Batch Effect Correction, and Principal Component Analysis (PCA). (A-B). Boxplots of integrated Gene Expression Omnibus (GEO) datasets before (A) and after (B) normalization. (C-D). PCA plots of integrated datasets before (C) and after (D) batch effect removal. (E-F). PCA visualization: E (2D) and F (3D) plots of sample distribution based on principal components. Blue = disease group (endometriosis, labeled "D"), yellow = healthy controls (labeled "H"). [file 41065_2025_590_MOESM1_ESM.jpg]

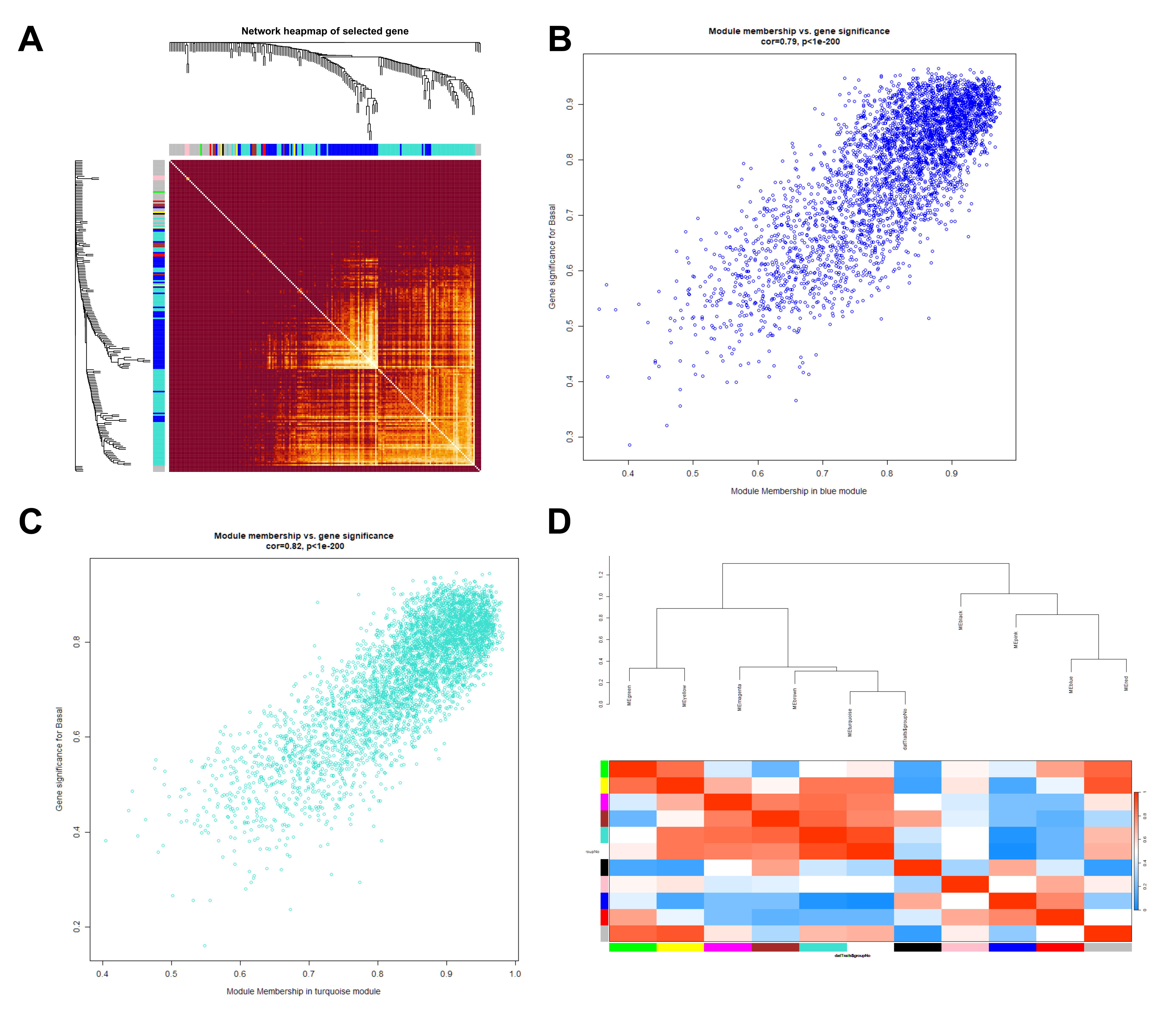

Supplement: Supplementary file 2 — Supplementary Material 2: Figure S2. (A). Network interconnection plot of selected genes. The figure illustrated the interaction network among selected genes. Each node represented a gene, and edges denoted connections or correlations between genes. (B-C). Gene significance vs. module membership scatter plots for the blue (B) and turquoise (C) modules. The plots displayed the significance of member genes within the module. The x-axis typically represented gene connectivity (i.e., the number of connections a gene has within the network), while the y-axis denoted gene significance (e.g., correlation with clinical traits such as disease status). (D). Dendrogram and heatmap of module correlations. The dendrogram depicted hierarchical clustering of modules based on gene expression patterns, while the heatmap visualized pairwise correlation coefficients between modules. Color intensity reflected the strength of correlations, with darker hues indicating stronger associations. [file 41065_2025_590_MOESM2_ESM.jpg]

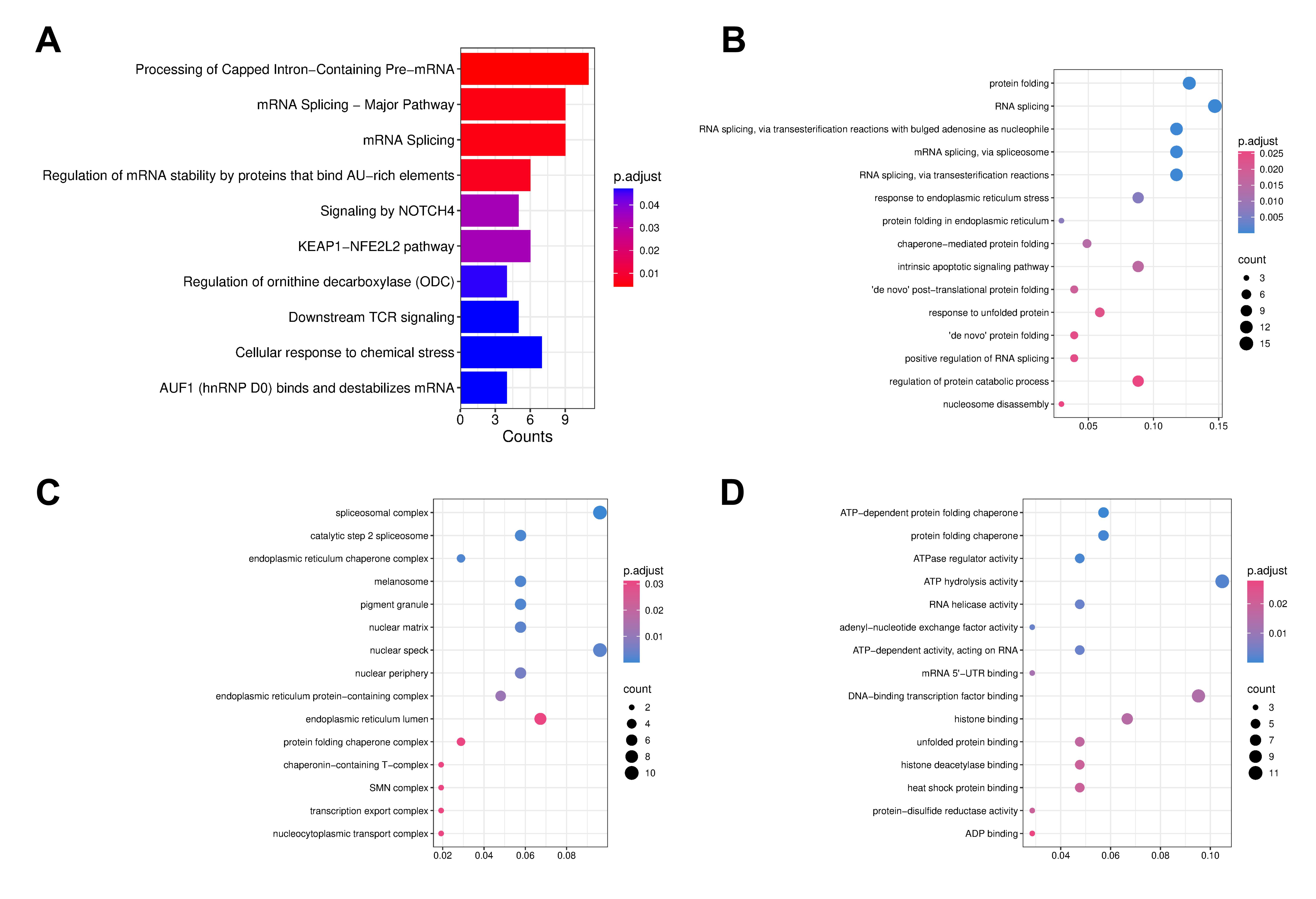

Supplement: Supplementary file 3 — Supplementary Material 3: Figure S3 (A). Reactome pathway enrichment analysis of candidate genes. (B-D). Gene Ontology enrichment analysis of candidate genes. (B) Biological Process (C) Cellular Component (D) Molecular Function. [file 41065_2025_590_MOESM3_ESM.jpg]

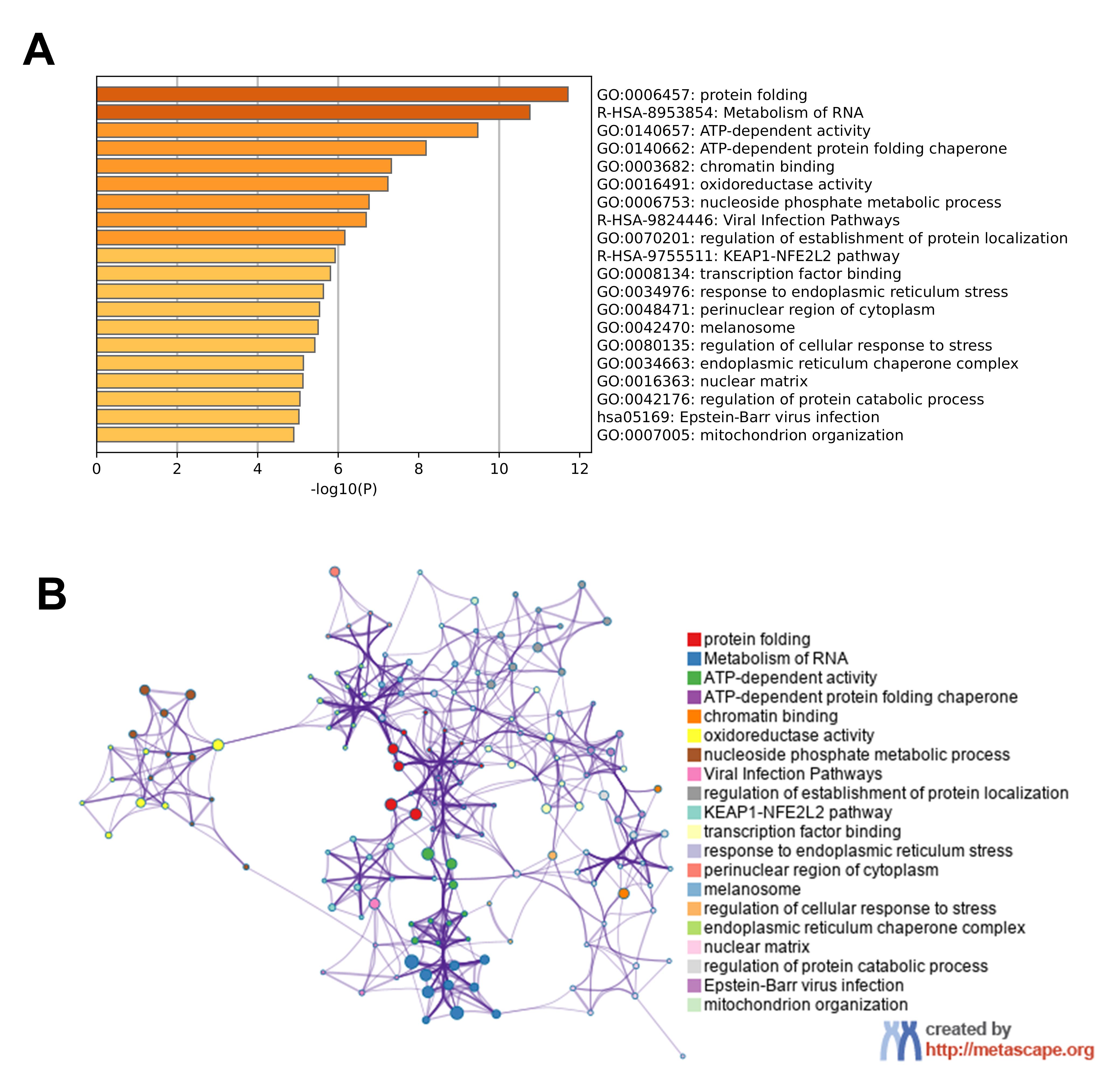

Supplement: Supplementary file 4 — Supplementary Material 4: Figure S4. A-B. Bar plot (A) and interaction network (B) of enriched pathways. [file 41065_2025_590_MOESM4_ESM.jpg]

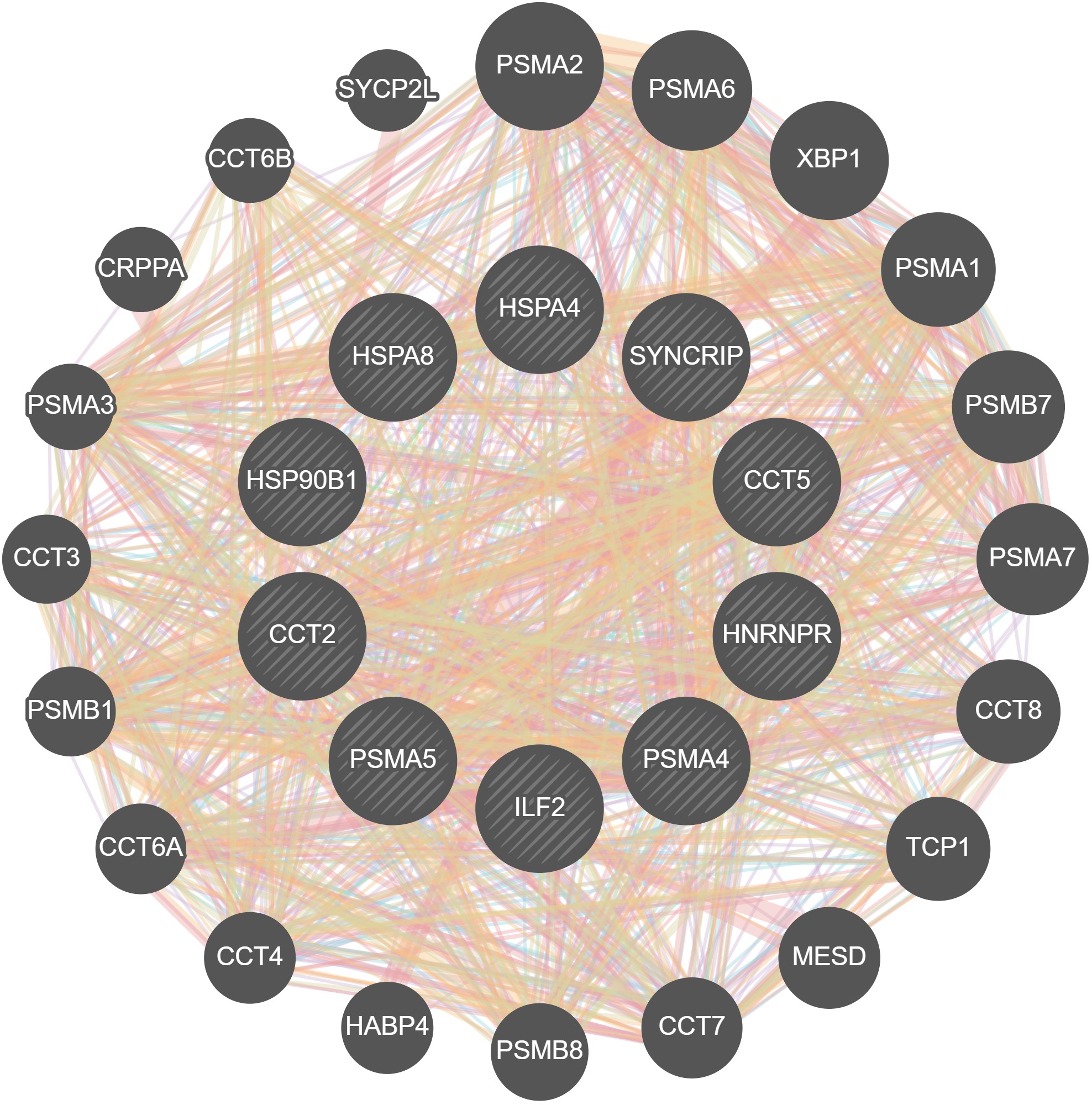

Supplement: Supplementary file 5 — Supplementary Material 5: Figure S5 Interaction network of hub genes generated by GeneMANIA. The network illustrates functional associations among hub genes and predicted related genes based on multiple interaction types. Line colors represent the type of supporting evidence: pink-purple: co-expression; pink: physical interactions; orange: predicted interactions; yellow: shared protein domains; blue: pathway co-participation; green: genetic interactions. [file 41065_2025_590_MOESM5_ESM.jpg]

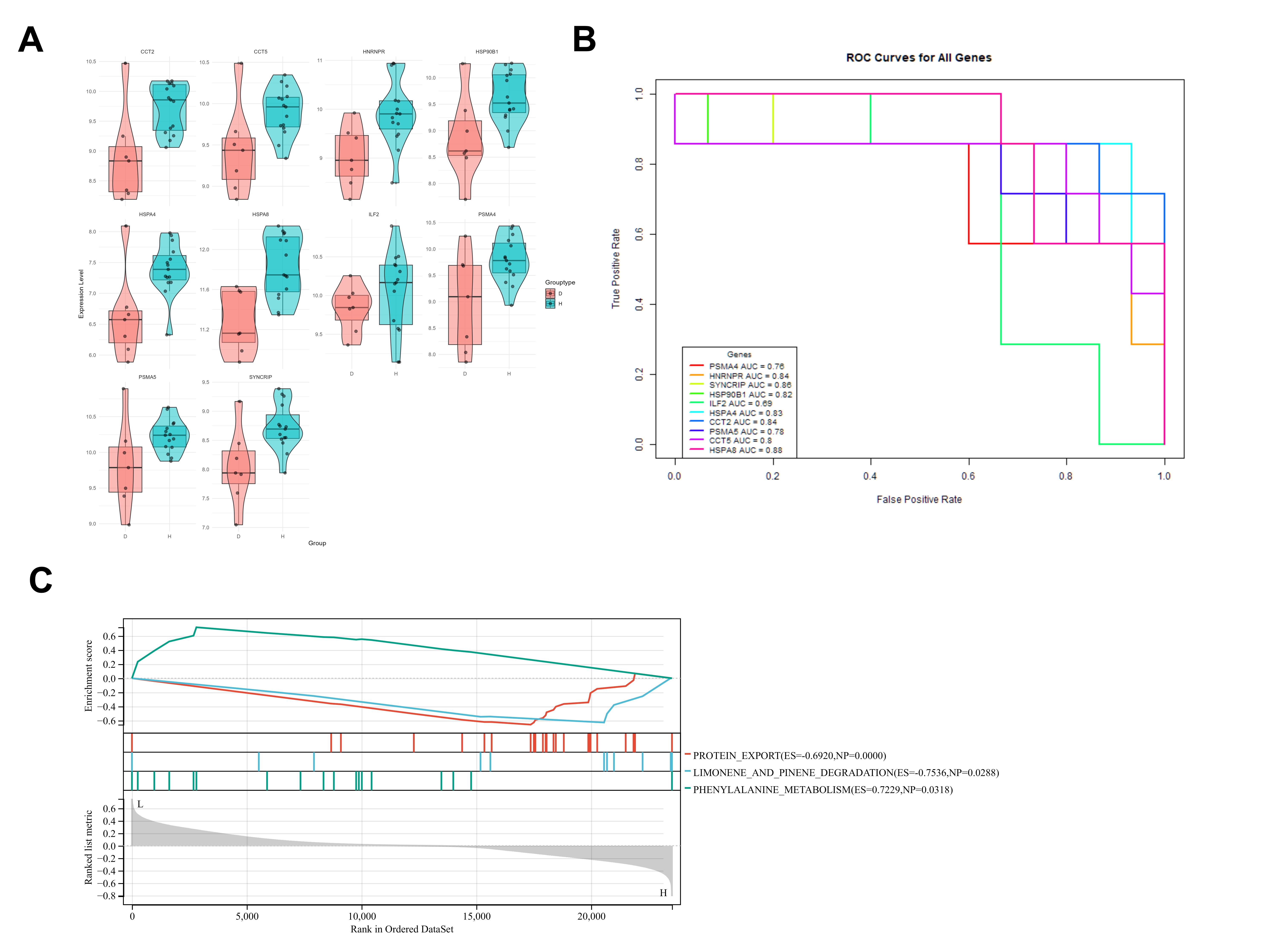

Supplement: Supplementary file 6 — Supplementary Material 6: Figure S6. Validation and Functional Exploration of Key Genes. A. Expression validation of key genes in the training set (GSE25628) via Wilcoxon test (P >0.05). B. Receiver operating characteristic curves and the area under the curve values for key gene diagnostic performance. C. Gene set enrichment analysis of HSP90B1 in the training set (top 10 pathways, P >0.05, FDR >0.25). [file 41065_2025_590_MOESM6_ESM.jpg]

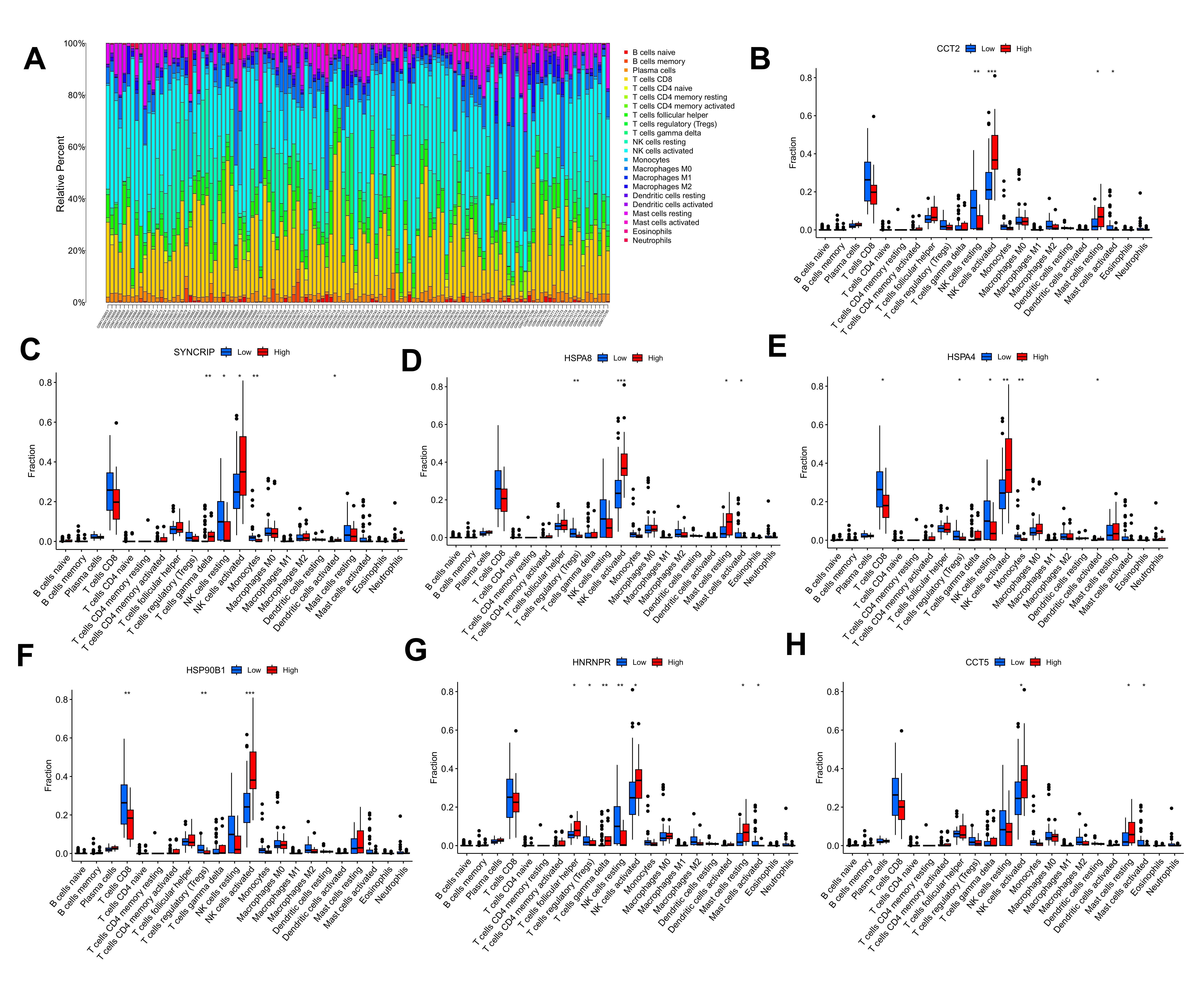

Supplement: Supplementary file 7 — Figure S7. Immune Infiltration Analysis. (A). Stacked bar plot of immune cell proportions in endometriosis vs. normal samples. (B-H). Correlation heatmaps between key genes and differentially infiltrated immune cells. (B) CCT2, (C) SYNCRIP, (D) HSPA8, (E) HSPA4, (F) HSP90B1, (G) HNRNPR, (H) CCT5. *P >0.05 ** P >0.01 *** P >0.001 vs. Low. [file 41065_2025_590_MOESM7_ESM.jpg]

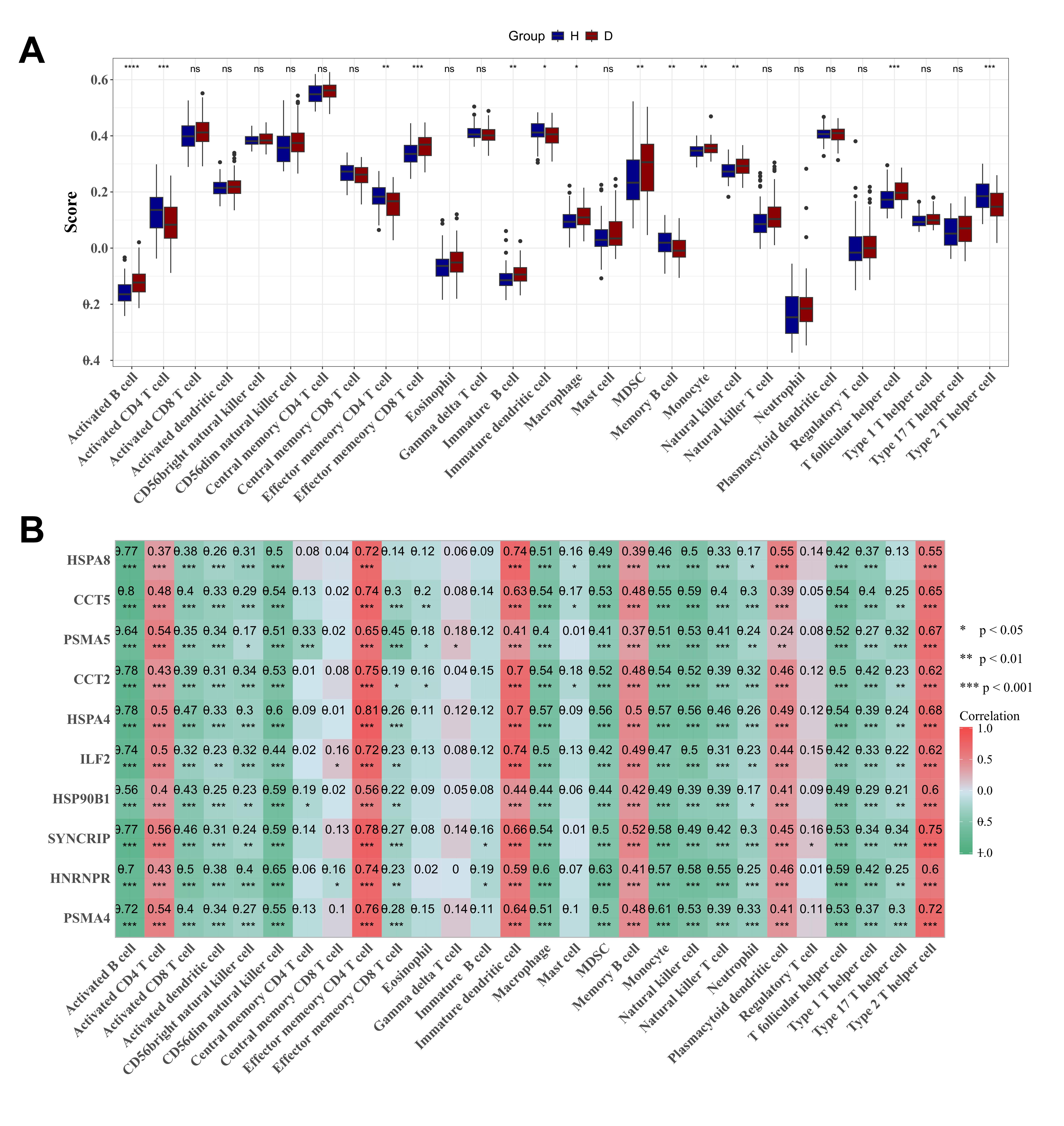

Supplement: Supplementary file 8 — Supplementary Material 8: Figure S8. Immune infiltration analysis based on ssGSEA. (A) Box plots showing the enrichment scores of 28 immune cell types between the disease and healthy groups in the training set. Statistical significance is denoted as follows: ns, not significant; *p >0.05, **p >0.01, ***p >0.001, ****p >0.0001. (B) Heatmap depicting the Spearman correlation coefficients between the expression levels of hub genes and the infiltration abundances of 28 immune cell types. [file 41065_2025_590_MOESM8_ESM.jpg]

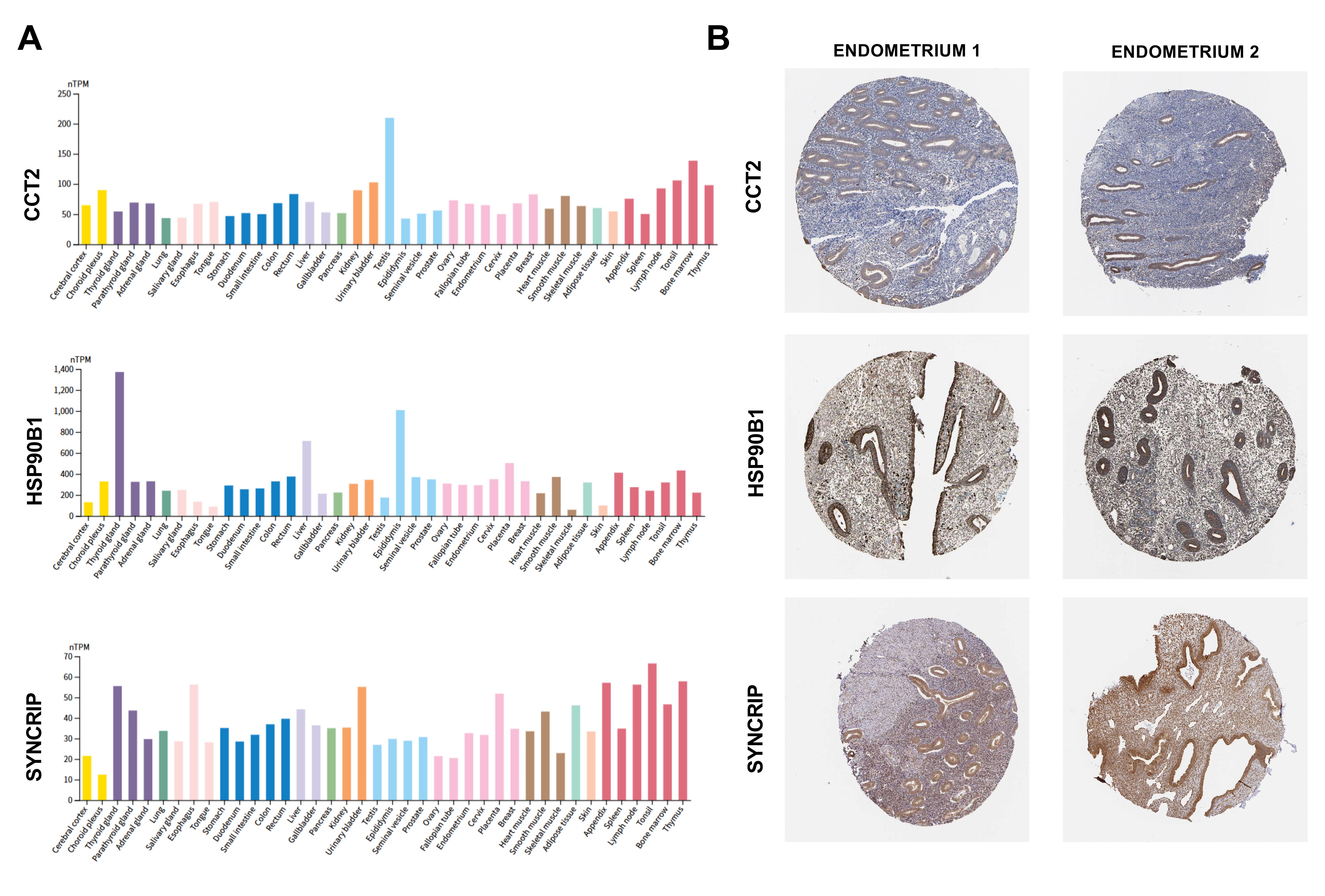

Supplement: Supplementary file 9 — Supplementary Material 9: Figure S9. Expression patterns of CCT2, HSP90B1, and SYNCRIP in normal human tissues. (A) Bar graphs showing protein expression levels of CCT2, HSP90B1, and SYNCRIP across various human organs, based on immunohistochemistry data from the Human Protein Atlas (HPA) database. (B) Representative immunohistochemical staining images of CCT2, HSP90B1, and SYNCRIP in two independent normal endometrial tissue samples, as retrieved from the HPA database. [file 41065_2025_590_MOESM9_ESM.jpg]

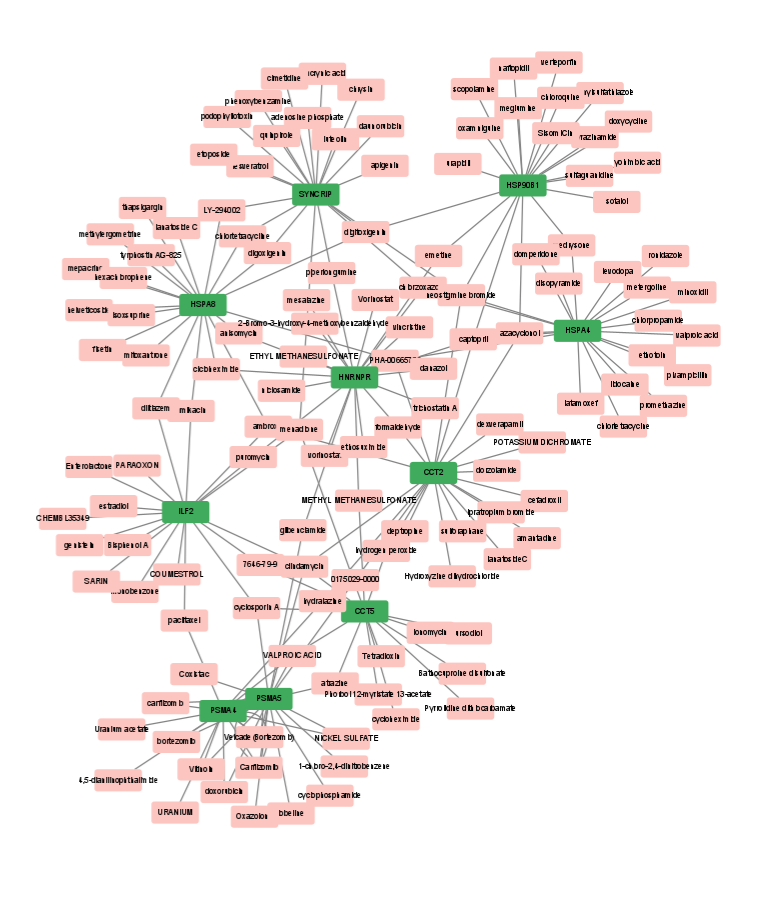

Supplement: Supplementary file 10 — Supplementary Material 10: Figure S10. Network of hub genes and their predicted potential drugs. The network illustrates interactions between the key genes (green nodes) and potential therapeutic compounds (pink nodes) identified through the DSigDB database. Edges represent predicted drug-gene associations. [file 41065_2025_590_MOESM10_ESM.png]
